# Improved accuracy for myocardial blood flow mapping with deep learning-enabled CMR arterial spin labeling (DeepMASL): Validation by microsphere in vivo

**DOI:** 10.1016/j.jocmr.2025.101989

**Published:** 2025-11-14

**Authors:** Ran Li, Caleb Berberet, Qi Huang, Pamela K. Woodard, Jie Zheng

**Affiliations:** Mallinckrodt Institute of Radiology, Washington University School of Medicine, St. Louis, MO, United States

**Keywords:** Cardiac magnetic resonance, Myocardial blood flow, Deep learning, Hyperemia, Coronary artery stenosis

## Abstract

**Background:**

Current myocardial arterial spin labeling (ASL) methods are sensitive to noise (background and physiology), which limits the accuracy of myocardial blood flow (MBF) measurement. In this study, we demonstrated a new deep learning-enabled myocardial ASL approach (DeepMASL) and evaluated its accuracy to quantify MBF in a canine model of coronary arterial disease in vivo. The reference method was invasive microsphere measurements.

**Methods:**

Eighteen mongrel dogs were divided into two groups: healthy (n = 9) and coronary stenosis (n = 9). The latter was induced in an open-chest model with 3 types of stenosis: 50% (n= 3), 70% (n = 3), and 90% (n = 3). Each dog received pharmaceutically induced hyperemia, by the infusion of either dipyridamole or dobutamine to induce different levels of MBF. Microsphere measurements were performed at rest and during the hyperemia. A cardiac ASL sequence was employed to acquire ASL signals at the mid-section of the heart, at rest and during the hyperemia. A physics-based deep learning network (DeepMASL) was developed using synthetic ASL signals with different levels of background noise. Segmented MBF values produced by both non-DeepMASL and DeepMASL methods were measured in all dogs to compare with segmented microsphere MBF values.

**Results:**

While the non-DeepMASL method severely underestimated hyperemic MBF by 33–49%, the DeepMASL approach dramatically improved the accuracy to obtain error less than 10%. There were strong correlations (*r* = 0.85 – 0.86) in segmented MBF values between measurements by DeepMASL and microsphere methods in either normal or ischemic dogs with varying degrees of coronary artery stenosis. The Bland-Altman analysis revealed mild to moderate variations of DeepMASL (95% confident interval: −1.3 to 1.5 ml/min/g in normal dogs and −1.8 to 1.3 ml/min/g in stenotic dogs) and almost zero bias.

**Conclusion:**

The novel DeepMASL demonstrates much improved accuracy in the quantification of regional MBF at varying levels of coronary artery stenosis, which is correlated strongly with microsphere MBF values. The validated data indicates the potential for this DeepMASL technique to be translated for noncontrast diagnosis of myocardial perfusion deficit in a clinical setting.

## Graphical abstract

**Figure:**
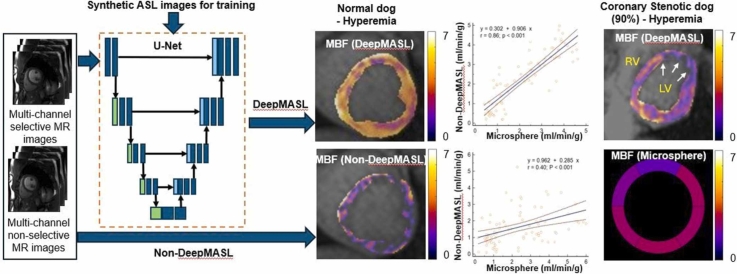

## 1 Introduction

Myocardial arterial spin labeling (ASL) method remains the only methodology in cardiovascular magnetic resonance (CMR) to quantify myocardial blood flow (MBF) in vivo, without using CMR contrast media. The method relies on the difference in myocardial signal intensity or T1 between using a slice selective and volume selective inversion recovery pulses [1], [2]. For this reason, the measurement of MBF is subjective to the errors associated with both background and physiological noises including motion artifacts and metabolic fluctuations [3], [4]. This problem is particularly prominent in the clinical setting at lower magnetic fields (≤ 3 T MRI systems). One manifestation of measurement errors is the non-uniform distribution of MBF map observed even in healthy myocardium [5].

A few efforts have been made to improve MBF map quality with suppression of noise levels by designing ASL sequences and/or optimizing image analysis methods [6], [7], [8], [9]. While such improvements were reported recently, myocardial ASL is still not ready for clinical translational study due to lack of robust performance comparing to the first-pass perfusion imaging method. In addition, very few studies had direct validation of the ASL measurements in the heart [5], [10]. In this project, a new deep learning approach based on a ASL physics model was developed and optimized for in vivo MBF measurements at a 1.5 T MRI system. A previous feasibility study demonstrated the potential of this specific ASL imaging for clinical translational study [11]. In this work, this deep-learning approach was tested in data sets obtained in a canine model of coronary artery diseases. The traditional microsphere measurements of MBF were used as the gold standard for the validation purpose.

## 2 Methods

### 2.1 Canine model

All animal protocols were approved by the Animal Studies Committee at the local institute (protocol # 20070166). Eighteen mongrel dogs (weight = 25.5 ± 3.6 kg) were divided into two groups: healthy (n = 9) and stenosis (n = 9). The later group was instrumented a coronary artery stenosis in the left anterior descending coronary artery (LAD). The procedure for the animal surgery and setting stenosis severity was described previously [12]. Briefly speaking, a thoracotomy was first performed in the fourth intercostal space and the pericardium incised. The LAD was dissected free distal to the first diagonal branch and then instrumented with a Doppler flow probe, a pneumatic occluder, and a CMR-compatible stenosis clamp. To setup different stenosis severity, serial 20-s occlusions were performed to delineate the hyperemic flow responses. After tightening the stenosis clamp, another occlusion was performed to assess the decrease in hyperemic flow. After attaining the desired level of stenosis defined by reduction in hyperemic flow, the occluder was removed. The dogs remained open-chest and were moved to the magnetic resonance imaging (MRI) suite. In the stenosis group, three types of stenosis were created: 50% (n= 3), 70% (n = 3), and 90% (n = 3). No thoracotomy surgery was performed in healthy control dogs. A catheter was inserted through the right femoral artery in each dog to withdraw reference blood samples for the measurement of MBF with microspheres.

Each dog was intubated with a 9.0 French eustachian tube and connected to a Harvard room air ventilator (Hugo Sachs Elektronik, March-Hugstetten, Germany). Anesthesia was maintained throughout the procedure with 5–10 cc of α-chloralose administered intravenously (IV) and titrated as needed. A six-element phased array coil was placed on the chest of the dogs for signal reception, and a body coil served as the transmitter. A four-lead patch was attached to the chest of each dog for ECG monitoring and pulse-sequence triggering.

### 2.2 Method for MBF measurement

The model to measure MBF was well established previously[14], in which myocardial T1 after volumetric selection (T_1,vs_) and slice-selective (T_1,ss_) IR pulses are used to estimate MBF using the follow equation [14].

(1) $$\mathrm{MBF}=\lambda \frac{T_{1,\mathrm{vs}}}{T_{1,\mathrm{blood}}}(\frac{1}{T_{1,\mathrm{ss}}}-\frac{1}{T_{1,\mathrm{vs}}})$$

where λ is the constant blood-tissue coefficient of water (0.92 ml/g); T_1,blood_ represents the average T_1_ of the left ventricular blood pool. It is readily to see that the accuracy of MBF measurement depends heavily on the accuracy of myocardial T_1_ calculation.

We implemented a cardiac ASL sequence to measure myocardial T_1,vs_ and T_1,ss_ to measure MBF in vivo [5]. Two sets of T1-weighted images after applications of a slice-selective and volume-selective inversion recovery (IR) pulses were obtained in a fashion of single-shot gradient-echo acquisition for each slice location. Each set consisted of 10 T1-weitghed images with a temporal resolution of one RR-interval. Similarly to the Look-Locker acquisition scheme [13], these images represented the multiple data points along the magnetization recovery time course after each IR pulse. The initial inversion time (TI) was the time from the middle of the inversion-recovery (IR) pulse to the time when the central k-space data of the gradient-echo acquisition was acquired. The rest of TI values were calculated as: initial TI + (n-1) x RR-interval, where n = number of T1-weighted images.

However, due to limited RR-interval time of the ASL sequence, a T1 saturation effect occurs between two consecutive T1-weighted image acquisitions during T1 recovery periods. A modified T1 fitting algorithm was developed based on the Bloch equation to account for this effect, described in detail previously [5]. In brief, assuming TR is the pulse repetition time of the gradient-echo acquisition with a flip angle of α, the signal intensity of S_N_ of central k-space in the Nth single-shot measurement after each IR pulse can be derived:

(2) $$\frac{S_{N}}{\sin(\alpha )}=A^{\frac{n}{2}-1}+M_{0}\times (1-e^{-\frac{\tau_{N}}{T_{1}}})\;+\;(A^{\frac{n}{2}}\times \cos(\alpha )\times e^{-\frac{\tau_{N}}{T_{1}}}+\frac{S_{N-1}}{\sin(\alpha )}\times B^{\frac{n}{2}}\times \cos(\alpha )\times e^{-\frac{\tau_{N}}{T_{1}}}){\times B}^{\frac{n}{2}-1}$$

Where M_0_ is the equilibrium magnetization.

n is the nth number of radiofrequency (RF) pulses within the single-shot gradient-echo acquisition, e.g., for 128 phase encoding lines, central k-space n = 64.

${\mathrm{TI}}_{N}$ is the time interval from the IR pulse to the central k-space signal $S_{N}$.

$$A^{\frac{n}{2}-1}=\;M_{0}\times (1-e^{-\frac{\mathrm{TR}}{T_{1}}})\times \frac{1-({\cos(\alpha )\times e^{-\frac{\mathrm{TR}}{T_{1}}})}^{\frac{n}{2}-1}}{1-\cos(\alpha )\times e^{-\frac{\mathrm{TR}}{T_{1}}}}$$

$$B=\cos(\alpha )\times e^{-\frac{\mathrm{TR}}{T_{1}}}$$

$$\tau_{N}=\{\begin{array}{c} {\mathrm{TI}}_{N}-\left(\frac{n}{2}-1\right)\times \mathrm{TR}\;(N=1)\\ {\mathrm{TI}}_{N}-{\mathrm{TI}}_{N-1}-\left(n-1\right)\times \mathrm{TR}\;(N\neq 1) \end{array}$$

The expression of signal in Eq. (2) accounts for the saturation effect induced by the multiple data acquisitions between each cardiac cycle for the T1 calculation.

### 2.3 CMR imaging

The CMR imaging session was performed on a 1.5-T clinical MR scanner (Siemens Healthineers, Erlangen, Germany). After scout image scanning to localize short-axis myocardial slice, the cardiac ASL sequence was employed to acquire ASL signals at the mid-section of the heart along the short-axis view. The ASL imaging parameters included: gradient-echo TR/TE = 2.4 ms / 1.3 ms, initial TI = 100 – 140 ms, flip angle = 5°, field of view = 220 ×138 mm^2^, slice thickness = 8 mm, and interpolated matrix size = 256 ×160. Each acquisition lasted approximately 15 s while the animal was held breath mechanically.

The ASL acquisition occurred at rest and during either dipyridamole induced vasodilation or dobutamine induced stress. Dipyridamole (Bedford Laboratories, Bedford, Ohio) was infused intravenously at a dose of 0.14 mg/kg/min for 4 min to cause vasodilation to increase MBF. Dobutamine (Hospira Inc., Lake Forest, Illinois) was started at 5 µg/kg/min and titrated at 5 µg/kg/min increments every 5 min until heart rate reached >130 beats/min (maximal dose to 30 µg/kg/min).

### 2.4 Radioactive and Color microsphere (MS)

In healthy control animals, radiolabeled microspheres, ^85^Sr and ^46^Sc, were injected as references for measuring MBF at the rest and during stress conditions, respectively [15]. In stenotic animals, nonradioactive color microspheres with a size of 10 µm, lanthanum and ytterbium, (STERIspheres; BioPhy Laboratory, Worcester, Massachusetts) were used for measuring MBF at rest and during the stress, respectively. All microsphere measurements occurred approximately 2–5 min after the CMR ASL measurements as described previously [16]. Following the completion of the MRI study, the dogs were sacrificed, and the hearts were excised. A slice of the heart at the image location was then cut into 16 or 32 pieces [15]. For radioactive microsphere measurements, radioactivity in each sample was counted on an LKB multichannel gamma well counter (LKB, Mount Waverly, Victoria, Australia). For color microsphere measurements, both heart tissue and reference blood samples were sent to the BioPhy Laboratory for assay. Results of counts from specific microspheres were compared with counts in the blood withdrawal syringe and MBF values in absolute terms (ml/g/min) were quantified by the standard reference technique [13].

### 2.5 DeepMASL

**Fig. 1:**
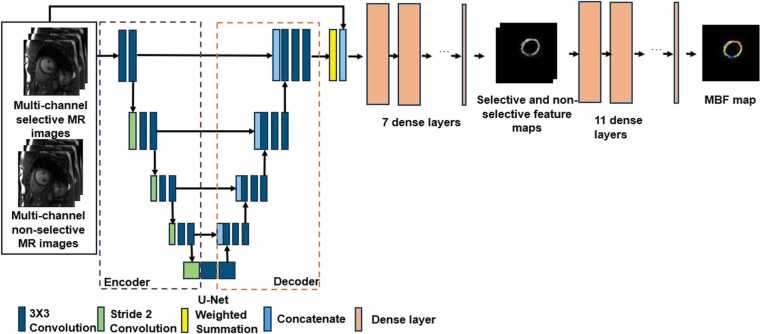
Illustration of a modified U-net structure for the DeepMASL model to calculate MBF. *DeepMASL* deep learning-enabled myocardial ASL, *MBF* myocardial blood flow

To quantify MBF, we developed a physics-based deep learning model, DeepMASL, trained using synthetic ASL signals with varying levels of added white noise. The DeepMASL model is based on a U-Net–style fully connected neural network comprising an encoder, a decoder, and a set of dense layers (Fig. 1). In the first stage of the network, the selective and non-selective ASL series were processed sequentially to generate corresponding feature maps. These maps were then concatenated and passed through additional dense layers to produce the final MBF maps.

Synthetic MR images and ground-truth MBF maps were generated using Eq. (1) and Eq. (2). First, synthetic MBF maps were created with mean values ranging from 0.2 to 7.0 ml/g/min and value-based standard deviations varying from 5% to 20% of the mean. To introduce spatial heterogeneity representative of normal and ischemic myocardium, we generated multiple normalized 2D Gaussian factor maps that produced smoothly varying multiplicative factors between 0.02 and 1. These factor maps were applied multiplicatively to the MBF maps to simulate regional flow variation. To impose realistic myocardial geometries, 100 left-ventricular myocardium ring masks manually segmented from real MR datasets were randomly applied to the synthetic MBF maps. These masks provided realistic LV geometry and spatial context for the network input.

**Fig. 2:**
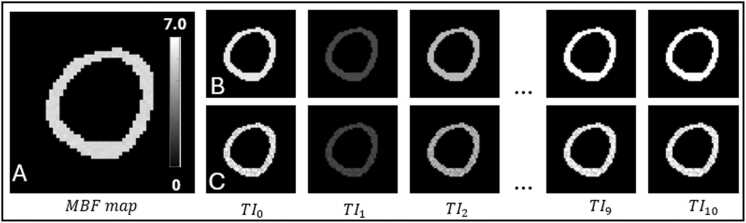
Illustration of synthetic stress MBF maps of normal myocardium (A) and corresponding synthetic selective MR image series without noise (B) and with Gaussian noise (SNR = 20) (C). The unit of MBF map is ml/min/g. *MBF* myocardial blood flow

Selective T₁ maps were modeled as Gaussian distributions with mean values ranging from 850 ms to 1100 ms and standard deviations between 20 ms and 80 ms, consistent with myocardial T₁ values at 1.5 T. Non-selective T₁ maps were then computed pixel-wise from Eq. (1) using the synthetic MBF and selective T₁ maps. Signal intensities for both selective and non-selective ASL images were calculated using Eq. (2), based on the corresponding MBF, selective T₁, and non-selective T₁ maps, in accordance with our imaging protocol. Figure 2A shows a stress MBF map of normal myocardium, and corresponding synthetic T_1_-weighted MR image series are shown in Figure 2B (no noise) and Figure 2C (noise added).

Finally, an intensity-based Gaussian noise model was applied to mimic realistic MR acquisition noise. Typical SNR levels were measured from real ASL data (SNR = 20–40), and similar Gaussian noise levels (2–5% of signal intensity) were added. Each synthetic dataset was generated with a random noise level within this range to improve network robustness. In total, 12,000 synthetic datasets were created, with 80% (9,600/12,000) used for training and 20% (2,400/12,000) for testing. The network was trained using mean absolute error (MAE) as the loss function.

### 2.6 Image and data analysis

Non-DeepMASL MBF maps were created by utilizing a custom-made software written in Matlab (MathWorks, Natick, Massachusetts), based on a previously established algorithm [5]. Myocardial masks were manually delineated and applied consistently for both the non-DeepMASL and DeepMASL methods to ensure comparable analysis regions. To suppress background noise and non-physiological values, a lower threshold of 0.01 ml/min/g was applied to the MBF maps. Each short-axis MBF map created by either DeepMASL or non-DeepMASL method was automatically divided into 6 segments based on the AHA segmentation model with the software by a single reader (CB). The corresponding microsphere MBF values in the same AHA segments were then obtained for the comparison. Bland Altman plots and Pearson’s correlation were used to compare MBF values between DeepMASL or non-DeepMASL and microsphere measurements. Linear mixed effect model was used to compare each outcome (microsphere, DeepMASL and non-DeepMASL) at rest or under stress, among four groups (normal, 50%, 70%, 90%) with random intercepts on dog and fixed effect of group. A p < 0.05 was considered statistically significance.

## 3 Results

**Fig. 3:**
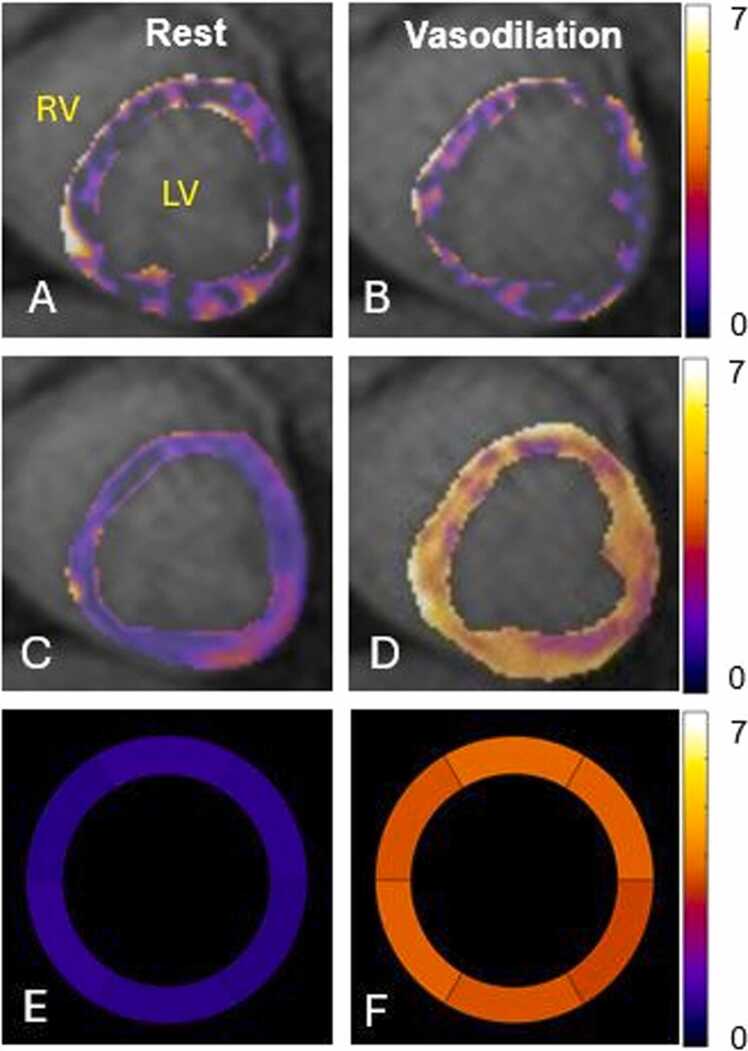
Examples of one pair of MBF maps (rest and vasodilation via dipyridamole infusion) obtained from a normal dog by using non-DeepMASL (A, B) and DeepMASL (C, D) approaches. Corresponding microsphere results in bullseye displays are shown in E and F. The unit of color bar scale is ml/min/g. *MBF* myocardial blood flow, *DeepMASL* deep learning-enabled myocardial ASL, *RV* right ventricle, *LV* left ventricle

**Fig. 4:**
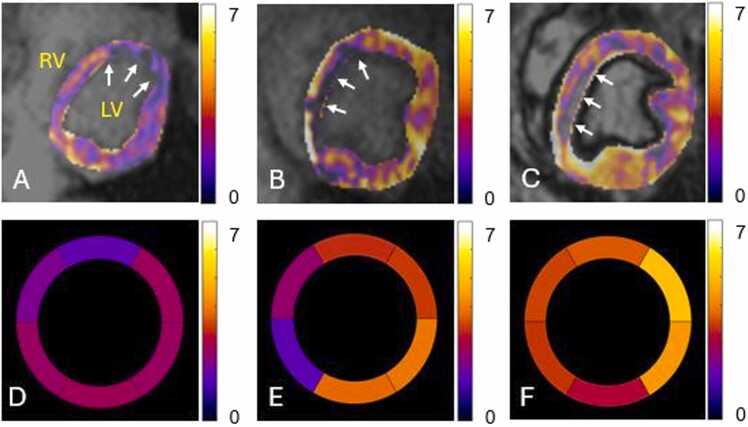
Three representative stress MBF maps created by DeepMASL are shown in A (stenosis 90%, dipyridamole), B (stenosis 70%, dobutamine), and C (stenosis 50%, dobutamine). The corresponding microsphere results in bullseye display are shown in D – F. The arrows indicate the perfusion deficits induced by various stenosis in anterior or septal regions. The unit of color bar scale is ml/min/g. *MBF* myocardial blood flow, *DeepMASL* deep learning-enabled myocardial ASL

**Fig. 5:**
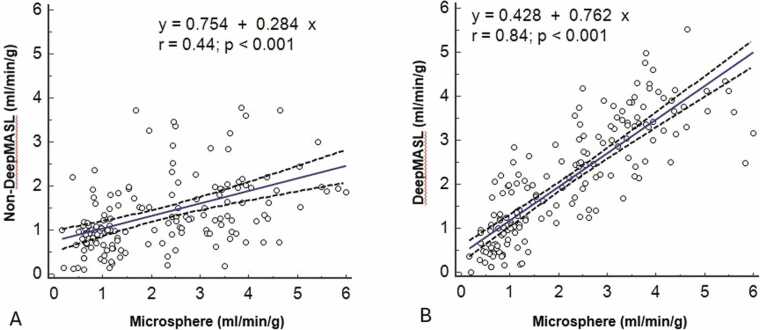
Correlations of MBF calculated by non-DeepMASL (A) and DeepMASL (B) versus microsphere MBF in all dogs. The two dashed curves represent 95% confidence intervals of the regression line (solid lines). *MBF* myocardial blood flow, *DeepMASL* deep learning-enabled myocardial ASL

**Fig. 6:**
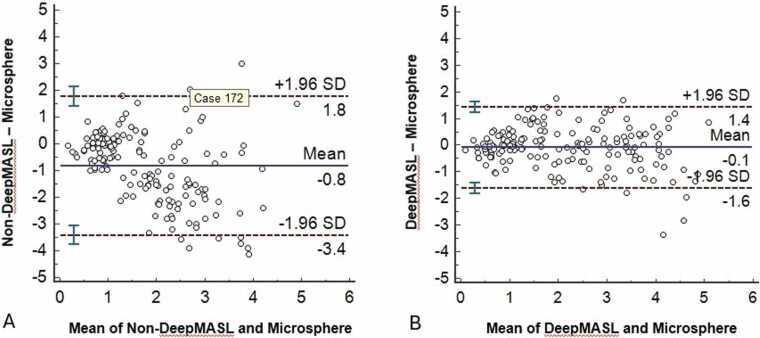
Bland-Altman plots to compare MBF measured by Non-deepMASL (A) and DeepMASL (B) versus microsphere methods in all dogs. *MBF* myocardial blood flow, *DeepMASL* deep learning-enabled myocardial ASL

Three sets of data (one set of rest and one set of stress data of two normal dogs and one set of stress data of a dog with 50% coronary artery stenosis) were discarded due to mishandling of some tissue or blood samples leading to incorrect microsphere measurements. Figure 3 and Figure 4 show examples of MBF maps measured in healthy dogs and dogs with varying coronary artery stenosis severity, respectively. There are moderate correlations in segmented MBF values between measurements by non-DeepMASL and microsphere methods in normal dogs (*r* = 0.56) and stenotic dogs (r = 0.4). With the use of DeepMASL, these correlations were strong (*r* = 0.85–0.86), although a miniscule bias was observed (Fig. 5). Bland-Altman plots are shown in Figure 6 to compare the DeepMASL and microsphere results in two normal and stenotic dogs, revealing mild to moderate variations of DeepMASL and almost zero bias.

**Fig. 7:**
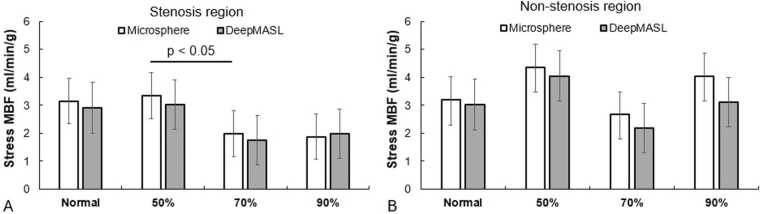
Comparison of absolute stress MBF measurements by microsphere (reference) and DeepMASL methods in myocardial regions perfused by stenotic coronary arteries (A) and by normal patent coronary arteries (B). No significant difference was observed. *MBF* myocardial blood flow, *DeepMASL* deep learning-enabled myocardial ASL

Figure 7 shows the comparison of mean stress MBF (DeepMASL and microsphere) in 4 groups of dogs in myocardial territories perfused by stenotic and normal coronary arteries. The mean stress MBF in stenotic regions with 50% coronary artery stenosis was maintained the same as in normal dogs. This value decreased apparently with 70% and 90% arterial stenosis, although significance was observed only in microsphere data between 50% and 70%. In non-stenotic regions, there were relatively large variations in stress MBF across different groups of dogs without any apparent trend. Table 1 summarize the major differences in mean MBF values of all myocardial segments observed in two groups of dogs, at rest and during stress.

**Table 1:** Mean CMR MBFs calculated from non-DeepMASL and DeepMASL, in comparison with microsphere results.

	Non-DeepMASL		DeepMASL		Microsphere
Normal Dogs (rest)	0.82±0.37		1.07±0.73		0.94±0.30
Normal Dogs (hyperemia)	1.68±1.48**		3.28±0.77		3.23±0.77
Stenotic Dogs (rest)	1.21±0.82*		1.38±0.96		1.57±1.49
Stenotic Dogs (hyperemia)	2.01±1.41**		2.72±1.12*		3.01±1.14

* p < 0.05, versus microsphere,

** p < 0.001, versus microsphere.

## 4 Discussion

We have previously developed a noncontrast ASL method in vivo to quantify MBF and demonstrated the feasibility in a canine model of coronary artery disease [5]. However, the image quality of the calculated MBF map was suboptimal. One major error source is the relatively high sensitivity of the ASL method to noise, including white random and physiology noise that may not have specific patterns. In this study, we proposed and validated a physics-based neural network model (DeepMASL) to create MBF maps by utilizing synthetic data sets, which had ground truth to be compared with. The result of considerable improvement in accuracy of MBF calculations over previous non-DeepMASL approach demonstrates the capability of DeepMASL to reliably measure MBF without using any contrast media. This is the first kind of study in vivo to validate a deep learning enabled cardiac ASL method that can be directly translated to clinical applications.

In conventional ASL methods, tissue perfusion calculations typically rely on sequential data from MR image series. This approach is highly sensitive to noise, motion artifacts, and other physiology noise. In brain studies, extensive research has been conducted on quantification of cerebral perfusion to address similar challenges by utilizing various deep learning approaches. For example, Hales et al. utilized an autoencoder model to suppress noise and transient artifacts in ASL MR images [17]. Gong et al. proposed a U-Net-based unsupervised deep learning method for denoising and reconstructing ASL MR images [18]. Shou et al. introduced a 3D transformer-based denoising network for ASL MR images [19]. While these methods significantly improve image quality, they focus solely on denoising and do not directly generate perfusion maps. Zhang et al. attempted to accelerate perfusion map reconstruction using a fully dense network on simulated dataset [20], but their method remains sensitive to noise and lacks consideration of spatial information. Furthermore, there are few studies addressing cardiac perfusion quantification using deep learning approaches. Our proposed DeepMASL method effectively integrates spatial and temporal information, which significantly enhances the accuracy, reliability, and robustness of MBF measurements. By employing a simulation-based approach, the method mitigates the challenge of limited ground truth data and ensures robustness to various noise levels.

Despite extensive research on cerebral perfusion, there is a significant gap in studies addressing cardiac perfusion quantification using deep learning approaches.

While DeepMASL permitted accurate measurements in normal dogs, mean MBF was generally underestimated in stenotic dogs, particularly during the stress conditions (Table 1). In addition to CMR measurement errors, there were several practical error sources contributing to the discrepancies between DeepMASL and microsphere. These include: 1) measurement times of ASL and microsphere were different, specifically in the stress MBF when MBF continued changing along the time course; 2) not precise co-registration of microsphere tissue samples and image segments; and 3) microsphere measurement errors [21], [22]. However, it is difficult to estimate the magnitude of each error contributing to the apparent error of MBF calculation. Therefore, limiting these error sources and reducing measurement errors remain critical for a successful MBF measurements with ASL approaches. Nevertheless, the percentage differences between MBF values determined by DeepMASL and microsphere are less than 15%, which is below the intra-subject variability > 26% [23].

## 5 Limitations

The current CNN-based architecture presents several primary limitations. First, the discrepancy between the synthetic dataset and real-world data may result in reduced output accuracy. Synthetic datasets, while valuable for controlled experimentation, may fail to capture the full complexity and variability of real-world imaging conditions. The variability of conditions may include imperfect RF flip angles, irregular ECG triggering, and respiratory motion. Future generation of completed synthetic data sets may need to consider such variability. Second, the reliance on fixed time intervals between consecutive images, i.e., fixed RR intervals, constrain the flexibility of MR imaging sequence design. This limitation affects the adaptability of the model to real-world scenarios where variable time intervals may be necessary due to arrhythmia. To overcome this, we plan to explore transformer-based architectures that can encode variable time interval information, enhancing the model's capacity for the MBF quantification. Third, the small sample size and varied imaging parameters, including the use of a 1.5 T MRI system, further limit the generalizability of our findings. A small dataset reduces the robustness of the model's predictions, as it may not adequately represent the diversity of data distributions in clinical populations. This limitation can lead to overfitting and biases, particularly when the model encounters out-of-distribution data. The variability in imaging parameters across datasets, such as differences in scanner settings or imaging protocols, poses additional challenges for model generalization. The reliance on a 1.5 T MRI system may not fully exploit the enhanced signal-to-noise ratio and resolution benefits offered by higher-field-strength systems such as 3 T.

There are several other experimental limitations in this methodology study. The sample size of stenotic dogs was small with only three dogs per stenosis degree. This may be the reason that significant differences in MBF values measured by DeepMASL were not detected among normal and stenotic dogs. Larger clinical studies, with established CMR first-pass perfusion imaging as reference, would provide an alternative approach to evaluate the accuracy and precision of the DeepMASL in the detection of myocardial perfusion ischemia. No physiological noise was added in the generation of synthetic data sets for training and testing. Consideration of modeling these noises may further improve the robustness of DeepMASL across diverse clinical challenges. Since myocardial and blood T₁ values differ across field strengths, retraining DeepMASL using simulations with field-specific T₁ and SNR parameters would be necessary to ensure optimal performance at other field strengths. We only scanned one slice in this study to match the slice of the myocardial tissue sample used for microsphere measurements. In clinical practice, multiple slices of perfusion maps would be obtained with multiple breath-holds. Another major issue is the motion control, especially in dogs with coronary artery disease under dobutamine stress. Arrhythmia was observed in these dogs, which lead to cardiac motion in some myocardial segments. With combined effects of relatively low SNR at 1.5 T MRI system and cardiac motion, heterogeneity of MBF maps was observed in some dogs (Figs. 3B and 3C). More sophisticated data acquisition and reconstruction will be needed to enable free-breathing multiple-slice ASL imaging in cardiac patients who cannot hold their breath and/or have severe arrhythmia.

## 6 Conclusion

This new DeepMASL approach demonstrates the accuracy for identifying regional differences in MBF with varying degrees of coronary artery stenosis in an experimental animal study, which is correlated strongly with microsphere MBF values. Since the imaging technique can be directly applied in human study, our data suggest that this technique has the potential to be translated to clinical applications for noncontrast diagnosis of myocardial perfusion deficit. More rigorous clinical evaluations will be needed before its full potential and clinical utility can be determined.

## Ethics approval

This animal project was approved by the Washington University Institutional Animal Care and Use Committee, and the approval number was 20010114.

## Funding

This work was supported in parts by NIH R01HL074019, R01HL165238, and American Heart Association (AHA) Second Century Implementation Science Award 23SCISA1145192.

## CRediT authorship contribution statement

**Pamela K Woodard:** Writing – review and editing, Visualization, Supervision, Resources, Project administration, Investigation, Funding acquisition, Data curation. **Qi Huang:** Writing – review and editing, Visualization, Validation, Methodology, Investigation, Formal analysis. **Caleb Berberet:** Writing – review and editing, Visualization, Validation, Investigation, Formal analysis, Data curation. **Ran Li:** Writing – review and editing, Writing – original draft, Visualization, Validation, Software, Methodology, Investigation, Formal analysis. **Jie Zheng:** Writing – review and editing, Writing – original draft, Visualization, Validation, Supervision, Resources, Project administration, Methodology, Investigation, Funding acquisition, Formal analysis, Data curation.

## Declaration of Competing Interest

All authors declare no competing interests for this study.

## Data Availability

The data will be available from the corresponding author on reasonable request.
